# Concentration-Dependent Exchange of Replication Protein A on Single-Stranded DNA Revealed by Single-Molecule Imaging

**DOI:** 10.1371/journal.pone.0087922

**Published:** 2014-02-03

**Authors:** Bryan Gibb, Ling F. Ye, Stephanie C. Gergoudis, YoungHo Kwon, Hengyao Niu, Patrick Sung, Eric C. Greene

**Affiliations:** 1 Department of Biochemistry and Molecular Biophysics, Columbia University, New York, New York, United States of America; 2 Department of Biological Sciences, Columbia University, New York, New York, United States of America; 3 Department of Molecular Biophysics and Biochemistry, Yale University School of Medicine, New Haven, Connecticut, United States of America; 4 Howard Hughes Medical Institute, Columbia University, New York, New York, United States of America; University of Iowa, United States of America

## Abstract

Replication protein A (RPA) is a ubiquitous eukaryotic single-stranded DNA (ssDNA) binding protein necessary for all aspects of DNA metabolism involving an ssDNA intermediate, including DNA replication, repair, recombination, DNA damage response and checkpoint activation, and telomere maintenance [1], [2], [3]. The role of RPA in most of these reactions is to protect the ssDNA until it can be delivered to downstream enzymes. Therefore a crucial feature of RPA is that it must bind very tightly to ssDNA, but must also be easily displaced from ssDNA to allow other proteins to gain access to the substrate. Here we use total internal reflection fluorescence microscopy and nanofabricated DNA curtains to visualize the behavior of *Saccharomyces cerevisiae* RPA on individual strands of ssDNA in real-time. Our results show that RPA remains bound to ssDNA for long periods of time when free protein is absent from solution. In contrast, RPA rapidly dissociates from ssDNA when free RPA is present in solution allowing rapid exchange between the free and bound states. In addition, the *S. cerevisiae* DNA recombinase Rad51 and *E. coli* single-stranded binding protein (SSB) also promote removal of RPA from ssDNA. These results reveal an unanticipated exchange between bound and free RPA suggesting a binding mechanism that can confer exceptionally slow off rates, yet also enables rapid displacement through a direct exchange mechanism that is reliant upon the presence of free ssDNA-binding proteins in solution. Our results indicate that RPA undergoes constant microscopic dissociation under all conditions, but this is only manifested as macroscopic dissociation (*i.e.* exchange) when free proteins are present in solution, and this effect is due to mass action. We propose that the dissociation of RPA from ssDNA involves a partially dissociated intermediate, which exposes a small section of ssDNA allowing other proteins to access to the DNA.

## Introduction

RPA is a heterotrimeric complex consisting of Rfa1 (70 kDa), Rfa2 (32 kDa), and Rfa3 (14 kDa), and the complex contains a total of six oligonucleotide/oligosaccharide (OB) folds, four of which are involved in ssDNA binding [3], [4], [5]. RPA binds tightly to ssDNA with a defined polarity and the four DNA-binding domains are termed dbdA, dbdB, dbdC, and dbdD [3], [4], [5], [6], [7], [8]. Rfa1 contains dbdA, dbdB, and dbdC, which are connected to one another by flexible linkers, and dbdD is found in Rfa2. RPA binds ssDNA in at least three distinct modes: a low affinity mode (K_d_∼100 nM) with a binding site size of ∼8 nucleotides, a moderate affinity mode (K_d_∼5 nM) with a binding site size of ∼12–23 nucleotides, and a high-affinity mode (K_d_∼0.05 nM) with a binding site size of ∼30 nucleotides [3], [4], [5]. In addition, *S. cerevisiae* RPA exhibits a salt-dependent transition from a binding site of ∼18–20 nucleotides to ∼26–28 nucleotides [9]. It has been suggested that these different binding modes may reflect the sequential association of distinctly ordered subsets of DNA-binding domains, which may facilitate initial binding to ssDNA as well as the displacement from ssDNA by other ssDNA-binding proteins [4].

RPA is essential for all aspects of DNA metabolism involving ssDNA intermediates, including homologous DNA recombination [1], [2], [3]. During homologous recombination the newly generated DNA ends are processed to yield long single-stranded DNA overhangs, which are then immediately bound by RPA [10], [11], [12], [13]. RPA protects ssDNA at processed DSBs from further enzymatic degradation, removes any secondary structure that could otherwise inhibit downstream steps in the repair pathway [3], serves as a DNA-damage checkpoint signaling intermediate [14], and recruits specific proteins to ssDNA through direct protein-protein interactions [1], [2], [10], [12], [15], [16], [17]. The Rad51 recombinase is required for both mitotic and meiotic DNA recombination, and is a member of the *RAD52* epistasis group, which also includes Rad50, Rad52, Rad54, Rad55, Rad57, Rad59, Rdh54 (Tid1), Mre11, and Xrs2 [18], [19], [20], [21]. The RPA-coated single-stranded DNA is the physiologically relevant substrate for the assembly of the Rad51 presynaptic filament [18], [19], [20], [21], and the presynaptic complex promotes initial pairing with and subsequent invasion of a homologous DNA template [18], [19], [20], [21], [22]. RPA also participates in later steps in the reaction by binding to the ssDNA strand that must be displaced from the homologous dsDNA template during strand invasion [23].

The RPA-ssDNA complex is the physiologically relevant target for presynaptic complex assembly, but paradoxically RPA can also prevent assembly of the presynaptic filament by inhibiting the binding of Rad51 to ssDNA. If added prior to or concurrently with Rad51, then RPA out competes Rad51 for available ssDNA binding sites [24], [25], [26], [27], [28], [29]. This effect can be overcome *in vitro* by adding RPA after Rad51, or through the inclusion of the recombination mediator protein Rad52 (in yeast), or Brca2 (in humans) [24], [25], [26], [27], [28], [29], [30], [31], [32]. *In vivo*, Rad52 helps load Rad51 onto ssDNA, allowing it to overcome the inhibitory effects of RPA [33]. Consistent with the view that RPA outcompetes Rad51 for ssDNA binding is the finding that mutations that strengthen RPA association with ssDNA make it more difficult for Rad51 to bind ssDNA (*e.g.* Rfa1 K45E) [34]. Conversely, mutations that increase Rad51 affinity for ssDNA partially overcome the need for mediator proteins, which would otherwise be necessary to promote binding on RPA-coated ssDNA (*e.g.* Rad51 I345T) [35]. Taken together, these studies imply that RPA prevents Rad51 association with ssDNA through a mechanism based on competitive inhibition.

To fulfill its biological function, RPA must be capable of binding very tightly to ssDNA, yet at the same time it must be readily displaced from ssDNA intermediates so that the ssDNA can be accessed by downstream proteins. This paradox is generally explained through a requirement for specific protein-protein interactions that help promote dissociation of RPA from ssDNA. For example, in the case of recombination this role is fulfilled by mediator proteins, such as Rad52 or BRCA2 that assist Rad51 loading on RPA-coated ssDNA. To help reveal insights into presynaptic complex assembly we begin looking at the behavior of *S. cerevisiae* RPA on ssDNA. We show that RPA can remain bound to ssDNA for hours at the infinite dilution limit, but when additional free RPA is present in solution the protein readily exchanges between free and bound states. Our results reveal an unanticipated dynamic exchange between ssDNA-bound RPA and free RPA solution, which allows RPA to bind ssDNA through a mechanism that can confer exceptionally slow off rates, yet also enables very rapid displacement of the protein through a direct exchange mechanism that is reliant upon the presence of free ssDNA-binding proteins in solution. This mechanism would ensure that ssDNA remains bound and protected by RPA, while at the same time allows RPA to be rapidly displaced from the ssDNA when necessary.

## Results

### DNA Curtain assay for RPA-eGFP-ssDNA filaments

**Figure 1:**
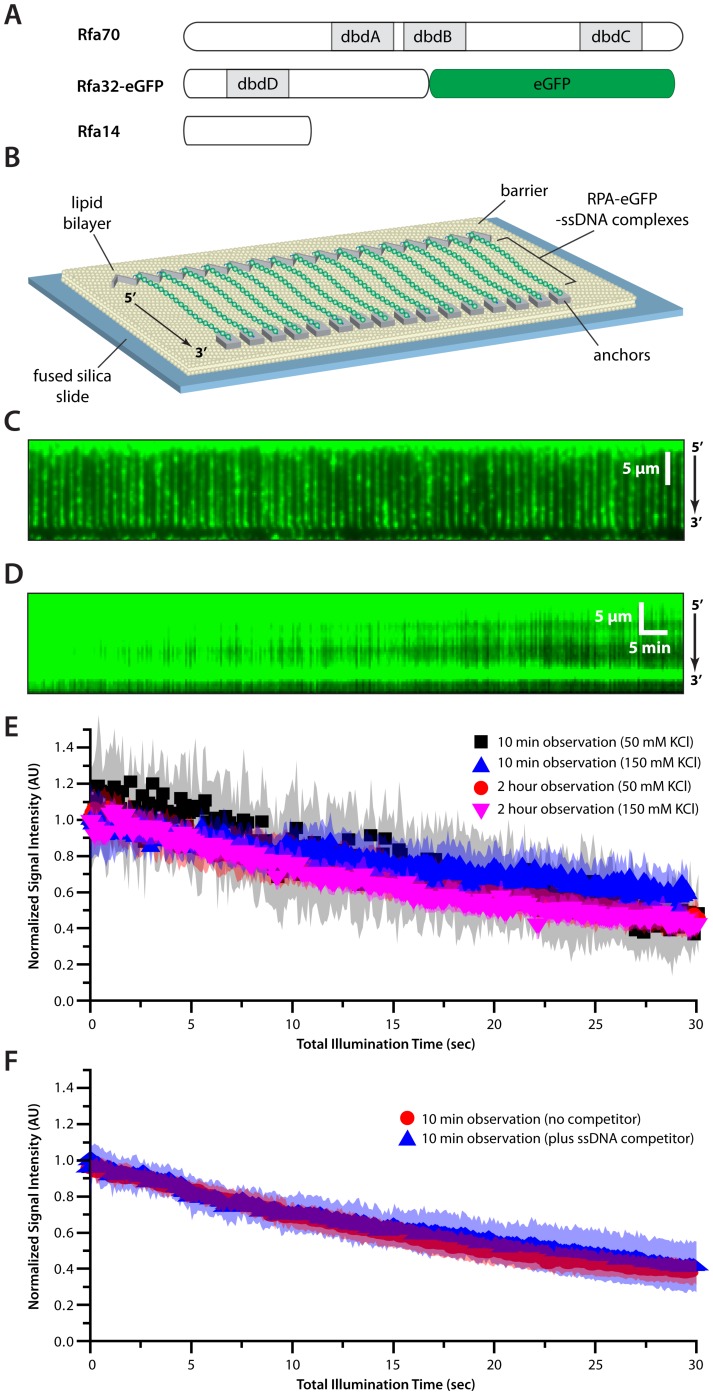
Single-stranded DNA curtain assay for RPA binding. (A) Schematic illustration of *S. cerevisiae* RPA showing the location of the four primary DNA-binding domains (dbdA-D) and the location of the eGFP tag at the C-terminus of RPA32. (B) Overview of RPA-ssDNA curtains showing the nanofabricated patterns on the surface of a fused silica microscope slide. All of the ssDNA molecules are anchored with their 5′ ends aligned along the leading edges of zig-zag shaped chromium (Cr) barriers [38], and their 3′ ends anchored through nonspecific adsorption to the exposed Cr pentagons, as depicted [40]. (C) Wide-field TIRF microscopy image of an ssDNA-curtain bound by RPA-eGFP. The 5′ to 3′ orientation of the ssDNA is indicated. Also see Video S1. (D) Kymograph showing a single RPA-eGFP/ssDNA complex with 100-msec images collected at 24-second intervals over a period of 2 hours. (E) Loss of RPA-eGFP signal is due to photo-bleaching. (F) Dissociation of RPA-eGFP is not accelerated in the presence of 1 µM competitor ssDNA. For both (E) and (F) intensity measurements for RPA-eGFP/ssDNA complexes viewed at 2-second intervals for a period of 10 minutes, or at 24-second intervals over 2 hours, as indicated. The total laser illumination period was the same under both experimental conditions. Each curve represents the normalized average calculated from 11–22 different ssDNA molecules collected at 50 or 150 mM KCl, and shaded regions correspond to the standard deviation for each data set.

We have established DNA curtains as a method for aligning large numbers of lipid-tethered DNA molecules at the leading edges of nanofabricated chromium (Cr) barriers within a microfluidic sample chamber where they can then be visualized by total internal reflection fluorescence microscopy (TIRFM)[36], [37], [38], [39]. Here we used DNA curtains to study the eukaryotic single-stranded binding protein RPA. For visualization, we used a fluorescent RPA construct in which eGFP (enhanced Green Fluorescent Protein) was fused to the C-terminus of the RPA2 32 kDa subunit (Figure 1A)[40]. This fusion protein is recruited to DSBs and retains full activity *in vivo* [33], and use of the RPA-eGFP fusion eliminated any need for the inclusion of a fluorescent DNA stain. We used rolling circle replication to generate long ssDNA substrates using a biotinylated oligonucleotide primer and circular M13 phage ssDNA as a template [40]. The resulting ssDNA was anchored to a fluid lipid bilayer within a microfluidic sample chamber and aligned along the leading edge of zig-zag shaped nanofabricated barriers by application of a hydrodynamic force (Figure 1B). The zig-zag barrier design allows the ssDNA molecules to be separated by a defined distance of at least 1 µm from one another [36], [38]. RPA-eGFP was injected into the sample chamber, and the downstream ends of the resulting RPA-ssDNA complexes were anchored through nonspecific adsorption to exposed Cr surfaces, allowing the eGFP-tagged complexes to be visualized by total internal reflection fluorescence (TIRF) microscopy in the absence of buffer flow (Figure 1C & Video S1)[40]. Unless otherwise stated, we utilized double-tethered ssDNA curtains for most experiments to minimize sample consumption.

Bulk biochemical data demonstrate that RPA can bind very tightly to ssDNA with sub-nanomolar affinities [3]. We have previously shown that RPA-eGFP remains stably bound to the ssDNA curtains for long periods of time (>60 minutes), and remains on the ssDNA even after injection of 3.5 M urea or 1 M NaCl [40]. These findings are fully consistent with previous bulk biochemical studies [3], highlighting the remarkable stability of the RPA-ssDNA complex. To further assess the lifetime of RPA-eGFP in our assays, we pre-assembled RPA-eGFP-ssDNA complexes in a sample chamber. All unbound protein was then quickly flushed out of the sample chamber, and the eGFP-labeled RPA-ssDNA complexes were monitored over either 10 minutes or 2 hours (Figure 1D); experiments beyond 2 hours are intractable due to stage drift and spontaneous breakage of the tethered ssDNA substrates. In both cases the RPA-eGFP signal decreased over time, but remarkably, the loss of RPA-eGFP signal per second of laser illumination time was identical for data collection windows spanning either 10 minutes or 2 hours (Figure 1E). Therefore the only observed change in the RPA-eGFP signal over time could be attributed to photo-bleaching of eGFP, and was not due to dissociation of RPA-eGFP from the ssDNA. RPA is necessary to remove secondary structure from ssDNA, and in the absence of RPA the ssDNA substrates used in our experiments remained highly compacted and cannot be stretched by application of buffer flow [40]. Dissociation of RPA-eGFP would therefore be expected to lead to a corresponding compaction of the ssDNA over time. We have previously shown that even though the RPA-eGFP signal decreases over time, this loss of fluorescence signal is not accompanied by a reduction in the apparent contour length of the ssDNA, providing further conformation that the change in signal is not due to dissociation of protein, but rather arises solely due to photobleaching [40]. In addition, the loss of RPA-eGFP signal was not accelerated in the presence of 1 µM ssDNA competitor, further suggesting that RPA-eGFP was not dissociating from the tethered ssDNA (Figure 1F). It should also be noted that the complexes were so stable that we were unable to determine the precise lifetime of RPA-eGFP bound to ssDNA in our assays because they greatly exceeded our data collection windows, but we can safely assert that the lifetime of the bound protein exceeds 2 hours under these reaction conditions. Taken together, our data shows that RPA-eGFP is highly resistant to dissociation from ssDNA, as expected based on bulk biochemical data [3].

### Rad51 stimulates rapid dissociation of RPA-eGFP from ssDNA

**Figure 2:**
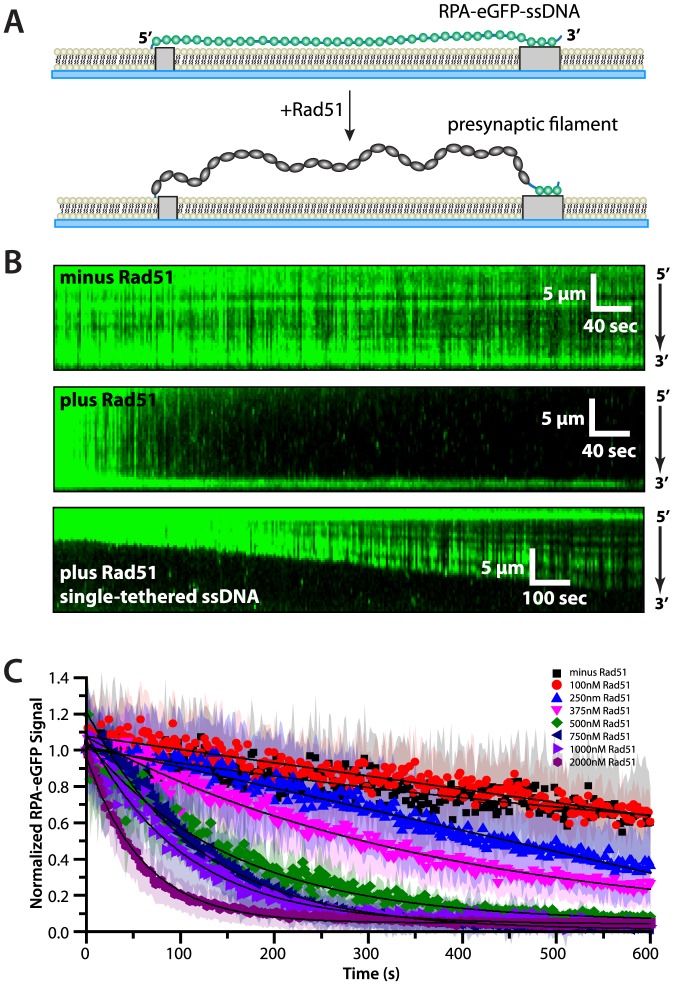
RPA-eGFP can be rapidly replaced from ssDNA by Rad51. (A) Schematic illustrating the predicted outcome for an ssDNA curtain experiment (side view) where RPA-eGFP is replaced by unlabeled Rad51. The loss of fluorescence as RPA-eGFP is displaced by Rad51 also coincides with an increase in the length of the ssDNA, which causes an increase in the transverse fluctuations of the ssDNA molecules. (B) The upper panel shows a kymograph of RPA-eGFP bound to ssDNA over time in the absence of Rad51, and the middle panel shows how RPA-eGFP is rapidly displaced from the ssDNA upon injection of 750 nM unlabeled Rad51 with 2.5 mM ATP. Also see Video S2. The lower panel shows an example of a single-tethered ssDNA molecule, which illustrates how Rad51 binding coincides with displacement of RPA-eGFP and extension of the ssDNA. This single-tethered measurement was made using 650 nM Rad51 and 1 mM ATP. (C) RPA-eGFP signal versus time collected at different concentrations of Rad51 (as indicated) in the presence of 2.5 mM ATP in buffer containing 50 mM KCl. Each curve represents the normalized average calculated from 11 to 70 different ssDNA molecules. Shaded regions correspond to the standard deviation for each data set. The data were fit to single exponential decays (solid lines), and loss of signal reflects a combination of photo-bleaching (as reflected in the minus Rad51 control), Rad51-induced dissociation of RPA-eGFP, and corresponding extension of the ssDNA, which causes the time-averaged position of the molecules to move further away from the surface.

We have previously used DNA curtain assays to visualize the assembly and disassembly properties of both human and *S. cerevisiae* Rad51, but these previous studies were all limited to the use of double-stranded DNA [41], [42], [43], [44]. However, the RPA-ssDNA complex is the physiologically relevant substrate for assembly of the Rad51-ssDNA presynaptic filament. Therefore we next asked whether the RPA-ssDNA substrates could support the assembly of wild-type Rad51 presynaptic complexes in the DNA curtain assay. We chose to use unlabeled Rad51 for these experiments because although GFP-tagged Rad51 is correctly targeted to DSBs *in vivo*, it is unable to complete downstream steps in the repair pathway [33]. Wild-type Rad51 was injected into the sample chamber, buffer flow was terminated, and the reactions were monitored over time in the absence of buffer flow. Under these conditions, assembly of a Rad51 presynaptic filament should be accompanied by a corresponding displacement of RPA-eGFP from the ssDNA, as well as extension of the ssDNA (Figure 2A). Therefore the loss of RPA-eGFP signal during presynaptic complex assembly is expected to arise from both the displacement of RPA from the ssDNA upon assembly of the Rad51 filament, as well as movement of the ssDNA filament out of the evanescent field due to the increased overall contour length (Figure 2A). RPA remained bound to the ssDNA in the absence of Rad51, as anticipated (Figure 2B, upper panel). In contrast, RPA-eGFP dissociated from the ssDNA when Rad51 was injected into the sample chamber, with more rapid RPA-eGFP dissociation observed at higher concentrations of Rad51 (Figure 2B, middle and lower panels, Video S2, and Figure 2C). Control experiments confirmed that the Rad51-dependent dissociation of RPA from ssDNA only occurred in the presence of ATP, and no RPA-eGFP dissociation was observed when ATP was omitted from the reactions (not shown), confirming that the assembly of the Rad51 filaments was ATP-dependent, as anticipated. These results show that the RPA-ssDNA complexes can be used as a substrate for assembly of presynaptic complexes comprised of unlabeled, wild-type Rad51.

As indicated above, these experiments utilized double-tethered DNA curtains, which allowed us to visualize RPA-eGFP displacement in the absence of buffer flow and helped minimize sample consumption. Rad51 binding is expected to increase the extension of the ssDNA by approximately 50% relative to a dsDNA molecule of the same length [21], [22], [45]. This change in length was accompanied by increased transverse fluctuations of the double-tethered ssDNA molecules during assembly of the Rad51 presynaptic filaments, although this effect is difficult to quantitate because it also coincides with loss of fluorescence signal as RPA-eGFP is displaced. To further illustrate that Rad51 binding lead to an increase in the length of the ssDNA we also conducted experiments under continuous buffer flow using single-tethered ssDNA curtains [36], [40], which confirmed that the ssDNA length increased as expected as Rad51 displaced RPA (Figure 2B, lower panel). Again, we were unable to visualize the fully assembled presynaptic filament comprised of wild-type Rad51 in these experiments due to concomitant loss of the RPA-eGFP signal. Nevertheless these experiments show that wild-type Rad51 binds to and extends the ssDNA substrate, as expected. Taken together, these findings further confirm that we are able to monitor assembly of wild-type Rad51 presynaptic filaments using ssDNA curtains based on the displacement of RPA-eGFP that accompanies the binding of Rad51 to ssDNA.

The finding that Rad51 could displace RPA-eGFP from ssDNA in the absence of mediator proteins was unanticipated, especially given that RPA alone could remain bound to ssDNA for hours at the infinite dilution limit (*i.e.* when there is no free protein present in solution). In addition, prior biochemical and genetic studies have clearly shown that Rad52 assists assembly of Rad51 filaments on RPA-bound ssDNA [24], [25], [26], [27], [28], [29], [33]. However, one crucial difference between our work and prior bulk biochemical or genetic studies is that we are able to flush free RPA out of the reaction mixture prior to the addition of Rad51, which allows us to directly assess Rad51-induced RPA-eGFP dissociation in the absence of any potential for RPA re-association. Moreover, close inspection of the prior bulk biochemical data reveal that although Rad52 does stimulate the assembly of Rad51 on ssDNA bound by RPA, this effect is negligible at high concentrations of Rad51 [25], which is consistent with our results. We conclude that Rad51 can directly stimulate the removal of RPA-eGFP from ssDNA in these assays.

### Rad51 filaments remain bound to the ssDNA when ATP is present

**Figure 3:**
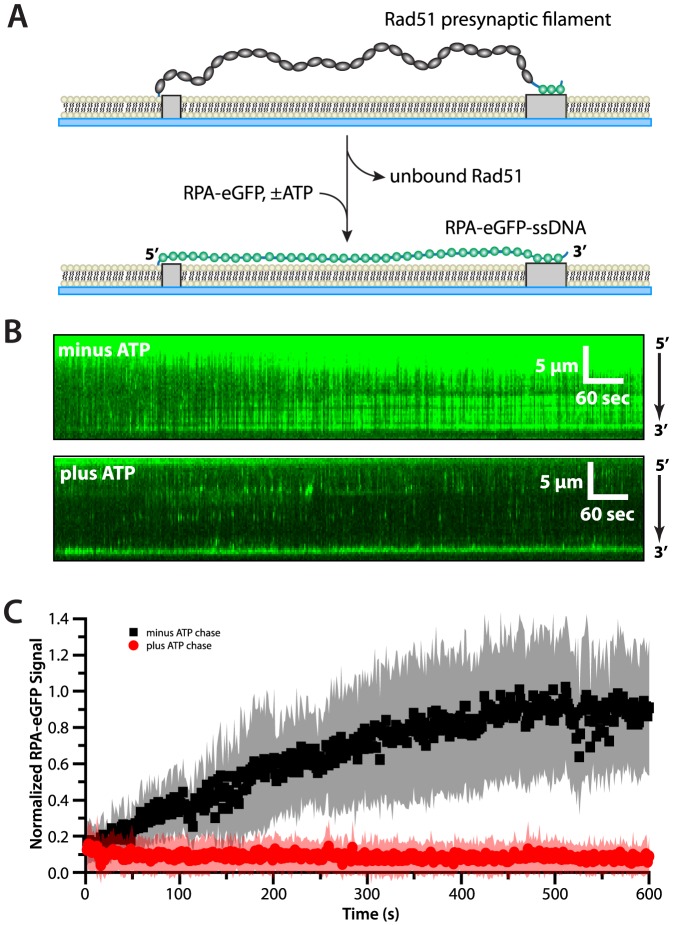
ATP prevents dissociation of Rad51 from ssDNA even when free RPA is present. (A) Experimental schematic illustrating the how replacement of wild-type, dark Rad51 with RPA-eGFP can be used to monitor disassembly of the presynaptic complex on double-tethered ssDNA curtains. (B) Examples of kymographs showing examples of wild-type Rad51 presynaptic complex disassembly reactions on single ssDNA molecules in the absence (upper panel) and presence (lower panel) of 2.5 mM ATP and 1 nM RPA-eGFP at 50 mM KCl. (C) RPA-eGFP fluorescence signal versus time during the Rad51 disassembly reactions. Each curve represents the normalized average calculated from 15 to 20 different ssDNA molecules, and shaded regions correspond to the standard deviation for each data set. When ATP is omitted from the chase buffer, the RPA-eGFP signal increases, reflecting the dissociation of Rad51 from the ssDNA. RPA-eGFP fails to bind to the ssDNA when 2.5 mM ATP is present in the chase buffer, indicating that Rad51 does not dissociate from the ssDNA.

We next used the ssDNA curtain assay to determine whether RPA might be capable of displacing Rad51 from ssDNA when there was no free Rad51 present in solution. For these experiments, unlabeled wild-type Rad51 was assembled onto the ssDNA substrates in the presence of ATP. Unbound Rad51 was then flushed from the sample chamber and quickly replaced with buffer containing 1 nM RPA-eGFP; these experiments where conducted at 1 nM RPA-eGFP to minimize the increased background signal arising from free RPA-eGFP at higher protein concentrations. Since there is no free Rad51 present in solution, the dissociation of Rad51 from the ssDNA should result in its replacement with RPA-eGFP, which is present in vast molar excess over any free Rad51 (Figure 3A). When the presynaptic complexes were chased with buffer containing no ATP, the Rad51 dissociated from the ssDNA with an observed half-life on the order of ∼3 minutes as revealed by the ability of RPA-eGFP to re-bind the ssDNA (Figure 3B–C). However, when the chase buffer contained 2.5 mM ATP, then Rad51 remained stably bound to the ssDNA in the presence of free RPA-eGFP, and we were unable to detect any appreciable dissociation of Rad51 over the time scales of these measurements (Figure 3B–C). These results demonstrate that the presence of ATP prevents displacement of Rad51 by RPA-eGFP.

### Concentration-dependent exchange of ssDNA-bound and free RPA

**Figure 4:**
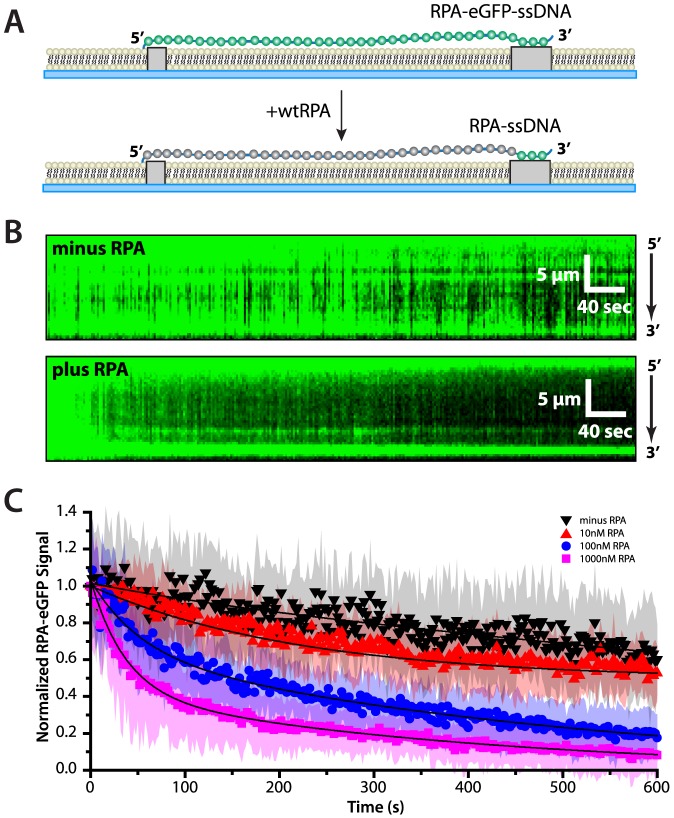
Concentration-dependent exchange of ssDNA-bound RPA. (A) Schematic illustrating the predicted outcome for an ssDNA curtain experiment (side view) where RPA-eGFP is replaced by unlabeled RPA. (B) The upper panel shows a kymograph of RPA-eGFP bound to ssDNA over time in the absence of free, unlabeled RPA, and the middle panel shows how RPA-eGFP is rapidly replaced upon injection of 1000 nM unlabeled RPA at 50 mM KCl. (C) RPA-eGFP signal versus time collected after the injection of different concentrations of unlabeled RPA (as indicated). Each curve represents the normalized average calculated from 15 to 33 different ssDNA molecules, and the shaded regions correspond to the standard deviation for each data set. The RPA chase data were fit to double exponential decays (solid lines), and loss of signal reflects a combination of photo-bleaching (as reflected in the minus RPA control), and unlabeled RPA-induced dissociation of RPA-eGFP, which increases at higher concentrations of free RPA. The minus RPA reference data set is that same as is shown in Figure 2C.

The finding that Rad51 alone could displace RPA from ssDNA, even in the absence of any mediator proteins, suggested the possibility that RPA might somehow be poised for displacement from ssDNA when other ssDNA-binding proteins are present in solution, regardless of their identity. To address this question further we next asked whether RPA-eGFP could be displaced from ssDNA by the addition of unlabeled, wild-type RPA (Figure 4). For these experiments, RPA-eGFP was first bound to the ssDNA, and then chased with varying concentrations of unlabeled RPA. Remarkably, these experiments revealed that ssDNA-bound RPA-eGFP was rapidly replaced when free wild-type RPA was present in solution (Figure 4B–C), despite the fact that RPA-eGFP remained tightly bound to ssDNA with a lifetime exceeding 2 hours when free RPA was not present in solution.

**Figure 5:**
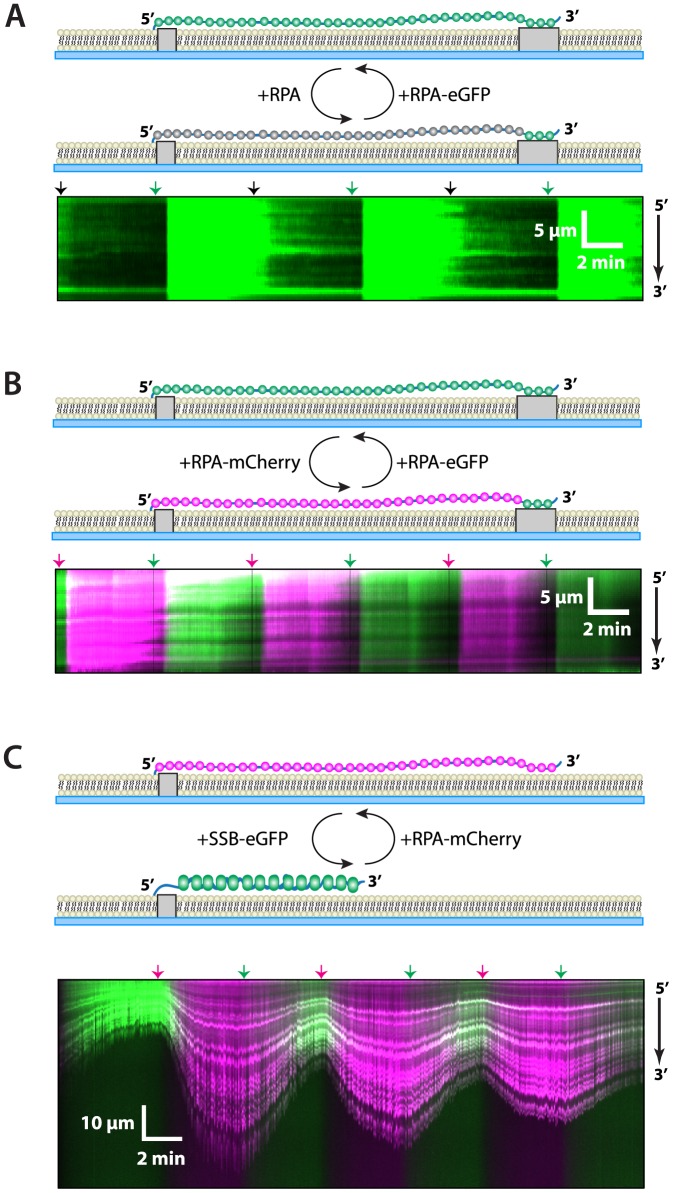
Two-color experiment showing exchange of bound and free RPA. Schematic depictions, kymographs, and graphs of exchange experiments conducted with alternating injections of (A) RPA-eGFP and dark, wild-type RPA, (B) RPA-eGFP and RPA-mCherry, or (C) RPA-mCherry (10 nM) and *E. coli* SSB-eGFP (40 nM). All reactions used buffer containing 150 mM KCl. Arrowheads placed above each kymograph indicate the time point of the protein injections, and are color-coded black, green or magenta to indicate dark protein, eGFP-tagged protein, or mCherry-tagged protein, respectively. The experiments in (A) and (B) used double-tethered ssDNA curtains, whereas the experiment in (C) used single-tethered ssDNA curtains to allow for the ssDNA compaction that accompanies the binding of SSB, as well as the corresponding extension that takes place when SSB is replaced by RPA. Also see Video S3.

The wild-type RPA chase experiments suggested that ssDNA-bound RPA could interconvert between the bound and free states, but only when additional free RPA was present in solution. As a further verification of this possibility, we next asked whether differentially labeled molecules of RPA could switch back and forth between free and bound states when sequentially injected into the sample chamber (Figure 5). We began by assembling RPA-eGFP on double-tethered ssDNA curtains, as described above. The RPA-eGFP/ssDNA complexes were then chased at approximately 5-minute intervals with alternating injections of 100 nM unlabeled wild-type RPA followed by 100 nM RPA-eGFP (Figure 5A). As shown here, at a protein concentration of 100 nM, RPA-eGFP could be replaced by wild-type RPA and vice versa, indicating that the protein was able to exchange between a free and bound state under these reaction conditions. This finding shows that the ability to undergo concentration-dependent exchange was not limited to the eGFP-tagged RPA, and that wild-type RPA also undergoes exchange when free proteins are present in solution. As a further conformation of our results, we next performed a two-color labeling experiment using alternating injections of 100 nM RPA-eGFP and 100 nM RPA-mCherry (Figure 5B and Video S3). These experiments confirmed that RPA could rapidly exchange between free and bound states so long as free RPA was present in solution as evidenced by the alternating colors of the two different colored proteins bound to the ssDNA.

### Eukaryotic RPA can exchange with bacterial SSB

Our findings show that ssDNA-bound RPA can be replaced when either Rad51 or additional free RPA are present in solution, but remains bound to ssDNA for hours at a time in the absence of free protein. This result raised the question of whether this outcome reflected a general property of RPA that did not depend upon the identity of the free ssDNA-binding protein in solution, or whether it only occurred when ssDNA-bound RPA was chased with either *S. cerevisiae* Rad51 or free RPA. For example, one possibility is that species-specific protein-protein interactions involving either RPA-Rad51 or RPA-RPA are necessary for the concentration-dependent dissociation of ssDNA-bound RPA. Alternatively, RPA might be replaced through mechanisms that do not necessarily require species-specific protein-protein interactions, but rather only require the presence of another ssDNA-binding protein in solution. We reasoned that if the ability to undergo concentration-dependent displacement was a general property of RPA, with no intrinsic requirement for specific protein-protein contacts, then a completely unrelated ssDNA-binding protein should also be able to promote the dissociation of RPA from ssDNA. To test this possibility, we next asked whether eGFP-tagged *E. coli* single-strand binding protein (SSB) could provoke the dissociation of *S. cerevisiae* mCherry-RPA from ssDNA. SSB is known to compact ssDNA upon binding, so these experiments utilized single-tethered ssDNA curtains to allow us to visualize changes in the ssDNA extension when switching back and forth between RPA-eGFP and SSB-mCherry (Figure 5C). As shown by these experiments, SSB could readily replace RPA bound to the ssDNA, and vice versa. These findings reveal that the displacement of ssDNA-bound RPA by free ssDNA-binding proteins does not require any species-specific protein-protein interactions and can even occur in the presence of a heterologous ssDNA-binding protein.

## Discussion

The ability to directly visualize the assembly of individual nucleoprotein complexes in real time offers a powerful approach for dissection of complex multi-component reactions pathways such as homologous DNA recombination. Here we have used total internal reflection fluorescence microscopy to visualize single-stranded DNA curtains bound by either RPA-eGFP or RPA-mCherry, and we use the displacement of these fluorescent versions of RPA as a read-out for the dynamic properties of RPA as well as the assembly of wild-type Rad51 presynaptic filaments. This system allows for temporally controlled delivery of reaction components, and recapitulates several known attributes of presynaptic filament assembly along with new, unanticipated behaviors of RPA.

The most striking finding from this study was that RPA can bind very tightly to ssDNA, with no detectable dissociation even over 2 hour observation periods, as expected based on bulk biochemical studies [3], but remains poised for rapid dissociation when other ssDNA-binding proteins are present in solution. Interestingly, very similar *in vitro* concentration-dependent turnover effects have recently been reported for the dsDNA-binding *E. coli* nucleoid proteins Fis and HU, the yeast HMGB protein NHP6A [46], and the *E. coli* restriction endonuclease EcoRI [47]. In all four cases the presence of free protein in solution dramatically increases the dissociation rate of the DNA-bound proteins [46]. Concentration-dependent dissociation has also been reported for bacterial SSB based on bulk biochemical measurements [48], and our single molecule assays also show that SSB displays concentration-dependent exchange when challenged with RPA. Together, these findings show that concentration-dependent exchange is not a unique property of eukaryotic RPA, but rather may be a general phenomenon that could extend to many other DNA binding proteins [48]. This possibility has profound implications for understanding how highly crowded physiological settings can impact protein turnover.

The unanticipated influence of free protein concentration on these dissociation processes has been interpreted to reflect microscopic dissociation that results in experimentally detectable macroscopic dissociation only when free proteins are present in solution [46]. Macroscopic dissociation means that a protein has completely dissociated from its substrate and has fully equilibrated with the surrounding solution. In contrast, microscopic dissociation means that a protein has dissociated from its substrate (either completely or partially), but has not yet equilibrated with the surrounding solution. During microscopic dissociation the protein comes off of the DNA, but only diffuses a short distance away from the molecule. Therefore microscopically dissociated proteins can immediately re-bind the DNA before equilibrating into solution so long as there are no other proteins present in solution to compete for re-binding [46]. However, when free proteins are present in solution they can compete for re-binding when DNA is made accessible due to a microscopic dissociation event. Therefore microscopic dissociation is only manifested as macroscopically detectable dissociation when other proteins are present to compete with the transiently unbound species for exposed DNA sites [46]. This distinction between microscopic and macroscopic dissociation is crucial for interpreting our results with RPA.

**Figure 6:**
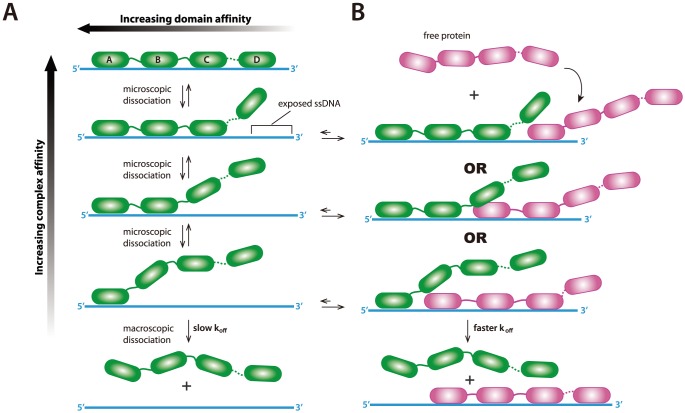
Hypothetical model for exchange-dependent dissociation of RPA from ssDNA. (A) Schematic illustration building on a previously proposed mechanism for binding and dissociation of RPA from ssDNA [4], and incorporating the concept of microscopic dissociation as a means of driving concentration dependent protein-exchange. During binding each of the four different DNA-binding domains (A to D) sequentially associates with the ssDNA. Intermediates involving submicroscopic dissociation of a subset of the DBDs still retain contact with the ssDNA and cannot macroscopically dissociate into solution, and in the absence of free protein each submicroscopic dissociation step is rapidly reversible. (B) When free ssDNA-binding proteins are present in solution, submicroscopic dissociation of any subset of the RPA DBDs will expose a small patch of ssDNA, providing the opportunity for the new proteins (shown in magenta) to bind the ssDNA. The presence of the newly bound protein will restrict re-association of the microscopically dissociated RPA DBD, thereby promoting macroscopic dissociation into solution of the original RPA molecule (shown in green).

As indicated above, Marko and colleagues have proposed that the concentration-dependent turnover of dsDNA-binding proteins occurs through a microscopically dissociated intermediate wherein a bound protein transiently dissociates from the DNA, but does not macroscopically dissociate back into free solution but rather rapidly rebinds to the same DNA site [46]. Similar mechanistic concepts can be applied to explain the concentration-dependent turnover kinetics we have observed for RPA. In the case of RPA, the phenomenon can also be extended to consider proteins with multiple DNA-binding domains where only subset of binding sites comes off of the DNA during a microscopic dissociation event, and it has previously been predicted that the dissociation of RPA from ssDNA might occur through exactly such a mechanism [4]. We propose a hypothetical model in which macroscopic dissociation of RPA into free solution is rendered extremely slow because the overall dissociation process is comprised of several, reversible microscopic steps involving each of the four individual ssDNA-binding domains (Figure 6A). However, when free ssDNA-binding proteins are present in solution they may engage any ssDNA that becomes transiently accessible when one (or more) of the RPA OB-folds microscopically dissociates from the substrate, which in turn would help provoke macroscopic dissociation of ssDNA-bound RPA by restricting re-association of microscopically dissociated domains (Figure 6B). In other words, mass action drives macroscopic dissociation of the microscopically dissociated intermediates. These findings are also akin to nucleosomes facilitating their own invasion, were nucleosome-bound dsDNA can transiently dissociate from the histone surface, and allow for the association of other dsDNA binding proteins [49], [50].

A crucial feature of this proposed RPA exchange-dependent dissociation mechanism is that it provides a means for RPA to directly “hand-off” ssDNA to downstream DNA processing enzymes while minimizing the potential for formation of secondary structure or exposure to nucleases. For example, a dissociation mechanism reliant upon the exchange-dependent displacement of RPA would ensure that unbound ssDNA does not exist long enough to either fold into new secondary structures or be degraded by nucleases, either of which would inhibit downstream reaction steps. However, our results show that RPA can even be exchanged for bacterial SSB, so we anticipate that any ssDNA binding protein might have the potential to access the ssDNA bound by RPA. This raises the question of how to prevent inappropriate exchange with other ssDNA-binding proteins present in the nucleus (*e.g.* ssDNA-binding nucleases), and regulate exchange such that correct proteins (*i.e.* Rad51) associate with the ssDNA? One possibility is that other HR proteins must bind to and regulate the exchange of RPA for proteins involved in downstream reactions steps. Alternatively, covalent modification of RPA may alter the exchange dynamics of ssDNA-bound RPA. Future studies will be necessary to evaluate these possibilities.

Our work shows that RPA can readily exchange between free and bound states *in vitro* through a concentration-dependent mechanism consistent with the existence of a microscopically dissociated intermediate, but this leaves the question of whether RPA might behave similarly *in vivo*. Importantly, live-cell fluorescence recovery after photo-bleaching (FRAP) experiments have shown that GFP-tagged RPA foci in mammalian cells display extremely rapid turnover *in vivo* [51], which contrasts dramatically with the exceedingly slow dissociation of RPA *in vitro*. It is tempting to speculate that the rapid *in vivo* turnover reflects the same or similar concentration-dependent exchange dynamics observed in our experiments. Moreover, the existence of concentration-dependent exchange involving RPA may be especially prevalent considering that *in vivo* concentrations of RPA are high (∼2 µM)[52], and local concentrations of RPA at repair foci or replication foci are expected to be significantly higher.

Collectively these studies start to suggest a general picture of protein-DNA complexes not as static entities whose constituent lifetimes can be defined by bulk biochemical data, but rather as highly dynamic assemblies where proteins are rapidly and readily exchanging between free and bound states through mechanisms dependent upon the presence (or absence) of free proteins. It should be noted that the most common bulk biochemical assays used to measure binding parameters typically rely upon a radiolabeled DNA substrate in combination with an unlabeled DNA competitor, and the proteins themselves are not labeled. These types of binding assays would fail to detect changes in dissociation rates that are dependent upon free protein concentrations because the nonspecific competitor DNA acts as a sink that essentially eliminates the population of free proteins, which in turn prevents free proteins from impacting the behavior of DNA-bound molecules. In addition, although most reports thus far of concentration-dependent protein-exchange have involved proteins that bind DNA with little or no sequence specificity, there is no reason to think that this phenomenon must be inherently restricted to this category of DNA-binding proteins. It is possible that concentration-driven protein dissociation from DNA may be much more prevalent than previously realized, and that these same concepts may also extend to site-specific binding proteins such as transcription factors.

Another seemingly surprisingly outcome of these experiments was that Rad51 alone was capable of rapidly displacing RPA from ssDNA with no need for any other mediator proteins, so long as free RPA is not present in solution. Rad51 and RPA compete for the same ssDNA binding sites, and RPA binds to ssDNA more tightly than Rad51 [24], [25], [27]. Under normal scenarios RPA binds to the ssDNA present at processed DSBs long before the arrival of Rad51 [23], [33], and the lifetime of the RPA-ssDNA is too long to allow for simple replacement by upon dissociation. As consequence, RPA can outcompete Rad51 for ssDNA binding both *in vitro* and *in vivo*, and Rad51 requires mediator proteins such as Rad52 [24], [25], [27], [33], implying that Rad51 itself lacks an intrinsic ability to remove RPA from ssDNA. In contrast to this prevailing view, our results show that Rad51 can remove and replace RPA from ssDNA when free RPA is absent from solution. If Rad51 alone can promote removal of RPA from ssDNA, how then might mediator proteins such as Rad52 act to promote assembly of Rad51 filaments on RPA-bound ssDNA? Moreover, given that heterologous exchange between *S. cerevisiae* RPA and *E. coli* SSB can occur *in vitro*, how are other ssDNA-binding proteins prevented from inappropriately accessing RPA (or SSB) bound ssDNA *in vivo*? Further work will necessary to determine the precise molecular basis for the influence of Rad52 on Rad51 presynaptic assembly, and whether other proteins such as Rad52 might influence the exchange behavior of RPA.

Finally, recent studies have suggested that dissociation of bacterial SSB during the assembly of RecA presynaptic complexes requires 1D diffusive motion along the ssDNA [53], [54]. However, these experiments were effectively performed at infinite dilution, which is in striking contrast with the *in vivo* situation where single-strand binding proteins such as eukaryotic RPA and bacterial SSB are typically among the most abundant proteins in the cell. We have no evidence either for or against 1D diffusion of RPA along ssDNA, but there is no need to invoke 1D diffusion to explain concentration-dependent protein exchange. We propose that a dominating influence driving macroscopic dissociation of RPA from ssDNA during Rad51 presynaptic complex assembly is concentration-dependent exchange of the proteins between the bound and free states. Our experiments using ssDNA curtains provide the basis for further investigations of the biochemical and biophysical properties of *S. cerevisiae* presynaptic complexes, and the influence that other *RAD52* group proteins have on the behavior of RPA and the assembly of the presynaptic filament, and also offer the potential for probing the later stages of recombination.

## Materials and Methods

### Proteins and DNA

Untagged *S. cerevisiae* Rad51 was purified as previously described [44]. 6xHis-tagged *S. cerevisiae* RPA RPA-eGFP and RPA-mCherry were expressed in *E. coli* strain BL21 as described [40]. In brief, cells were harvested, resuspended in lysis buffer (50 mM NaKPO_4_, 250 mM NaCI, 10 mM imidazole [pH 7.9]), and lysed by sonication. The clarified lysate was bound to Ni-resin (Qiagen) then washed with buffer containing 50 mM NaKPO_4_, 250 mM NaCI, and 20 mM imidazole. The protein was eluted with 50 mM NaKPO_4_, 250 mM NaCI, plus 250 mM imidazole, and dialyzed against 2 L of 30 mM Tris [pH 8.0], 1 mM DTT, 0.25 mM EDTA, 0.01% NP40, 80 mM NaCl. The protein was purified further by MonoQ (GE Healthcare) with a linear gradient of 80–700 mM NaCl, as described [40]. RPA-eGFP was dialyzed overnight against 1 L of buffer (30 mM Tris [pH 8.0], 150 mM NaCl, 1 mM DTT, 0.5 mM EDTA). The purified protein was concentrated with polyethylene glycol (PEG; Thermofisher), dialyzed against storage buffer containing 50% glycerol, frozen in liquid N_2_ and then stored at −80°C. The final RPA-eGFP or RPA-mCherry concentrations were determined from the absorbance of the eGFP or mCherry chromophores at 488 nM (ε_488 nm_ = 55,000 cm^−1^M^−1^) or 587 nm (ε_587 nm_ = 72,000 cm^−1^M^−1^), respectively [55].

A plasmid (p11d-tscRPA) encoding all three subunits of wild-type (non-fluorescent) *S. cerevisiae* RPA was a generous gift from Dr. Marc Wold [56]. The genes encoding RPA1, RPA2, and RPA3 were sequenced, and 7 mutations were corrected to ensure that the genes matched the sequences in the yeast genome database. An AvaII site was then introduced at the 3′ end of RPA2 via inverse PCR mutagenesis, and PCR insert derived from the plasmid pTXB3 (New England Biolabs) was inserted into the AvaII site. This construct (p11d-tscRPA-30MxeHis6) allowed RPA to be expressed as fusion construct tagged with an intein, chitin binding domain, and 6xHis tag at the C-terminus of RPA2. Wild-type RPA was then expressed in *E. coli* BL21DE3, 6 L of cells were grown at 37°C, and induced overnight at 16°C with the addition of 0.5 mM IPTG. Cells were harvested by centrifugation, resuspended into 35 ml of lysis buffer (50 mM NaKPO_4_, 250 mM NaCl, 10 mM imidazole), plus EDTA free protease inhibitor cocktail (0.5 mM AEBSF, 10 µM E-64, 2 mM Benzamidine), and 1 mM PMSF. Cells were then lysed by sonication, and the clarified lysate was bound to 6 ml of Ni-NTA resin (Qiagen) for 1 hour in batch. The bound resin was pelleted, resuspended into lysis buffer, and then poured into a column and washed with 80 ml of Ni-wash buffer (50 mM NaKPO_4_, 250 mM NaCl, 20 mM imidazole). RPA was then eluted with 15 ml Ni-elution buffer (50 mM NaKPO_4_, 250 mM NaCl, 250 mM imidazole), and the eluate was applied to a column containing 12 ml of chitin resin (New England Biolabs). The column was washed with 180 ml of chitin wash buffer (20 mM Tris-HCl [pH 8.0], 250 mM NaCl, 0.1 mM EDTA), and then exchanged into chitin wash buffer containing 50 mM DTT and allowed to cleave for 16 hours at 4°C. The cleaved protein was then eluted, concentrated in a Slide-a-lyzer cassette (7 kDa MWCO) with PEG concentrating solution (Thermo Scientific, Cat. No. 66528), and finally dialyzed into RPA storage buffer (50% glycerol, 20 mM Tris-HCl [pH 8.0], 150 mM NaCl, 0.1 mM EDTA). Protein concentrations were determined by SDS/PAGE with Coomassie staining and comparison to a BSA standard, and 20 µM aliquots were flash frozen on liquid N_2_ and stored at −80°C.

The gene for *E.coli* SSB was PCR amplified from genomic DNA. eGFP DNA was PCR amplified using primers containing homology to SSB and BamH1 at the 5′ end, and a streptactin tag followed by XhoI site at the 3′ end of the gene. Gene splicing by PCR was used to generate the final SSB-eGFP-streptactin tag gene product, which was cloned into a modified pETDuet vector (Novagen) and transformed into BL21-DE3 cells. A starter culture from a single colony was used to inoculate 6 liters of LB + ampicillin and grown at 37°C. Upon reaching an optical density of 0.6, the culture was induced with IPTG to 0.5 mM and grown for 20 hours at 18°C. Cells were harvested by centrifugation, resuspended in strep buffer (25 mM Tris−HCl [pH 7.4], 500 mM NaCl, 5% glycerol, 1 mM EDTA) supplemented with a protease inhibitors (0.5 mM AEBSF, 10 µM E-64, and 2 mM Benzamidine) and lysed by sonication. The clarified lysate was applied to a 5 ml streptactin sepharose gravity column (IBA life sciences) and washed with 300 ml strep buffer. SSB-eGFP was eluted in 20 ml strep buffer containing 2.5 mM desthiobiotin and concentrated with PEG 20,000 to 2 ml. The protein was then dialyzed into storage buffer (50 mM Tris-HCl [pH 7.4], 300 mM NaCl, 50% glycerol) and stored at −80°C. The concentration was determined by measuring the absorbance of eGFP.

Single-stranded DNA substrates were generated by rolling circle replication, as described [40]. In brief, single-stranded M13mp18 (NEB) was annealed to a biotinylated primer, and excess primer was removed by passage through a size exclusion spin column (Princeton Separations). Replication reactions contained 50 mM Tris [pH 7.4], 2 mM DTT, 10 mM MgCl_2_, 10 mM ammonium sulfate, 0.15 nM primed M13mp18 DNA (Invitrogen), and 200 µM dNTPs in a total volume of 100 µL. Reactions were initiated by addition of φ29 DNA polymerase to a final concentration of 100 nM and incubated for 30 minutes at 30°C, as described [40]. Reactions were terminated by the addition of EDTA to a final concentration of 75 mM.

### Single-stranded DNA Curtains

Chromium barriers were fabricated on fused silica microscope slides using electron-beam lithography, as described [36]. In brief, slides were first cleaned in NanoStrip (CyanTek Corp), rinsed with acetone and isopropanol and dried with N_2_. Slides were spin-coated with two layers of polymethylmethacrylate (PMMA; 25K and 495K; MicroChem), followed by a layer of Aquasave (Mitsubishi Rayon). Patterns were written with a FEI Sirion scanning electron microscope (J. C. Nabity, Inc.). Aquasave was removed with deionized water and resist was developed using isopropanol:methyl isobutyl ketone (3:1) for 1 minute with ultrasonic agitation at 5°C. The substrate was rinsed in isopropanol and dried with N_2_. Barriers were made with a 15–20 nm layer of chromium (Cr), and following lift-off, samples were rinsed with acetone and dried with N_2_.

Flowcells and lipid bilayers were prepared as described [36], [40]. Briefly, vesicles comprised of DOPC (1,2-dioleoyl-sn-glycerophosphocholine), 0.5% biotinylated-DPPE (1,2-dipalmitoyl-snglycero-3-phosphoethanolamine-N-(cap biotinyl)), and 8% mPEG 550-DOPE (1,2-dioleoyl-sn-glycero-3-phosphoethanolamine-N- [methoxy(polyethylene glycol)-550]) were deposited onto the sample chamber. The surface was then rinsed with Buffer A [40 mM Tris-HCl (pH 7.4), 1 mM DTT, 1 mM MgCl_2_, 0.2 mg ml^−1^ BSA]. The ssDNA was coupled to the bilayer through a biotin-streptavidin linkage and aligned at the barriers by application of buffer flow [40].

#### Reaction Conditions, Data Acquisition and Analysis

Experiments were performed using a prism-type TIRF microscope (Nikon) with two back-illuminated iXon EMCCDs (Andor Technology). For one-color experiments, illumination was provided by a 200 mW, 488-nm laser, as described [40]. For two-color experiments, illumination was provided by a 200 mW, 488-nm laser and a 200 mW, 561-nm laser (Coherent, Inc.). Intensity at prism face was ∼14 mW and ∼25 mW for the 488-nm and 561-nm lasers, respectively. Fluorescence signals were separated by a filter cube equipped with a dichroic mirror (ZT561rdc), band pass filter (ET525/50m), and long pass filter (ET575lp)(Chroma Technology Corp.).

For visualizing the RPA-ssDNA complexes, RPA-eGFP (0.2 nM) was first injected at an initial rate of 1.0 ml min^−1^ in buffer containing 40 mM Tris-HCl (pH 7.4), 1 mM DTT, 1 mM MgCl_2_, 0.2 mg ml^−1^ BSA. Unbound RPA-eGFP was then flushed from the sample chamber, buffer flow was terminated, and the ssDNA molecules were located by visual inspection. Unless otherwise stated, all subsequent reaction steps were conducted at 30°C in buffer containing 30 mM Tris-acetate (pH 7.5), 5 mM Mg-acetate, 50 mM or 150 mM KCl (as indicated), 1 mM DTT, 2.5 mM ATP and 200 µg ml^−1^ BSA [25]. The ssDNA competitor assay used a 70-mer oligonucleotide competitor (1 µM; 5′-CTC TCA GGG CCA GGC GGT GAA GGG CAA TCA GCT GTT GCC CGT CTC ACT GGT GAA AAG AAA AAC CAC CCT G -3′), which was injected into the sample chamber immediately after flushing away unbound RPA-eGFP. Rad51-induced & RPA-induced RPA-eGFP dissociation measurements were preformed by initiating data collections while quickly injecting the indicated amount of wild-type Rad51 or wild-type RPA. Buffer flow was turned off, 100 msec images were captured at 2-second intervals, and data collection continued for a period of 10–15 minutes. RPA exchange experiments were performed with alternating injections of either 100 nM wild-type (dark) RPA, RPA-eGFP, or RPA-mCherry, as indicated. Disassembly of the Rad51 presynaptic filament was measured by first binding Rad51 (4 µM) to an RPA-eGFP ssDNA curtain in the presence of 2.5 mM ATP. Rad51 binding was verified by loss of the RPA-eGFP signal. The buffer containing free Rad51 and ATP was then replaced with buffer containing 0.1 nM RPA-eGFP plus or minus 2.5 mM ATP, as indicated. All data used to generate kymographs and integrated signal intensity graphs were measured over an 11-µm segment of the ssDNA between the upstream barriers and the downstream anchor points. For quantitation, all data was normalized, corrected for background using a region of the slide surface without any ssDNA, and each trace represents average of at least 10 to 70 different ssDNA molecules.

## Supporting Information

Video S1 **Wide-field TIRFM image of RPA-eGFP bound to a double-tethered ssDNA curtain.** The ssDNA curtain was made using 1 µM zig-zag barriers, which were used to control the distance the adjacent ssDNA molecules. The ssDNA is unlabeled and the protein is shown in green.
(MOV)

Video S2 **Displacement of RPA-eGFP by Rad51.** RPA-eGFP bound to ssDNA curtains was chased with an injection of 100 nM Rad51 in the presence of 2.5 mM ATP. Buffer flow was turned off after Rad51 was injected into the sample chamber and the reactions were visualized over time.
(MOV)

Video S3 **Two-color visualization of RPA exchange.** ssDNA curtains were initially prepared with RPA-eGFP, and then visualized while performing alternating injections of 100 nM RPA-eGFP (shown in green) and 100 nM RPA-mCherry (shown in magenta).
(MOV)
